# Experimental evidence of pharmacological management of anchorage in Orthodontics: A systematic review

**DOI:** 10.1590/2177-6709.20.5.058-065.oar

**Published:** 2015

**Authors:** Felipe José Fernández-González, Aránzazu Cañigral, Felipe Balbontín-Ayala, José Manuel Gonzalo-Orden, Felix de Carlos, Teresa Cobo, Jose Pedro Fernández-Vázquez, Fernando Sánchez-Lasheras, José Antonio Vega

**Affiliations:** 1 Professor, University of Oviedo, Postgraduate program in Orthodontics and Dentofacial Orthopedics, Asturias, Spain; 2 Professor, University of Valencia, Postgraduate Program in Orthodontics and Dentofacial Orthopedics, Valencia, Spain; 3 Private practice, University of Andes, Chile.; 4 Full Professor and Chair, University of León, School of Veterinary Medicine, Department Surgery and Radiology, Léon, Spain; 5 Professor, University of Oviedo, Department of Surgery and Medical-Surgical Specialties, Asturias, Spain; 6 Private practice, University of Oviedo, Spain; 7 Department of Construction and Manufacturing Engineering, University of Oviedo; 8 Full professor and Chair, University of Oviedo, School of Medicine, Department Morphology and Cell Biology, Asturias, Spain

**Keywords:** Tooth movement, Orthodontic anchorage procedures, Osteoprotegerin, Bisphosphonates, Anti-inflammatory agents

## Abstract

**Introduction:**

Orthodontic anchorage is one of the most challenging aspects of Orthodontics. Preventing undesired movement of teeth could result in safer and less complicated orthodontic treatment. Recently, several reviews have been published about the effects of different molecules on bone physiology and the clinical side effects in Orthodontics. However, the effects of local application of these substances on the rate of orthodontic tooth movement have not been assessed.

**Objectives:**

The aim of this research was to analyze the scientific evidence published in the literature about the effects of different molecules on orthodontic anchorage.

**Methods:**

The literature was systematically reviewed using PubMed/Medline, Scopus and Cochrane databases from 2000 up to July 31^st^, 2014. Articles were independently selected by two different researchers based on previously established inclusion and exclusion criteria, with a concordance Kappa index of 0.86. The methodological quality of the reviewed papers was performed.

**Results:**

Search strategy identified 270 articles. Twenty-five of them were selected after application of inclusion/exclusion criteria, and only 11 qualified for final analysis. Molecules involved in orthodontic anchorage were divided into three main groups: osteoprotegerin (OPG), bisphosphonates (BPs) and other molecules (OMs).

**Conclusions:**

Different drugs are able to alter the bone remodeling cycle, influencing osteoclast function and, therefore, tooth movement. Thus, they could be used in order to provide maximal anchorage while preventing undesired movements. OPG was found the most effective molecule in blocking the action of osteoclasts, thereby reducing undesired movements.

## Abstract (pt)

**Introdução:**

a ancoragem ortodôntica é um dos aspectos mais desafiadores da Ortodontia. A prevenção de movimentos dentários indesejados poderia resultar em um tratamento ortodôntico mais seguro e menos complexo. Recentemente, foram publicadas várias revisões de literatura sobre os efeitos de diferentes substâncias na fisiologia do tecido ósseo e os efeitos colaterais clínicos na Ortodontia. Porém, os efeitos da aplicação local dessas substâncias no grau de movimentação dentária ortodôntica não foram avaliados.

**Objetivos:**

o objetivo da presente pesquisa foi analisar a evidência científica publicada na literatura sobre os efeitos de diferentes substâncias na ancoragem ortodôntica.

**Métodos:**

a literatura foi sistematicamente revisada utilizando-se as bases de dados PubMed/Medline, Scopus e Cochrane, de 2000 a 31 de julho de 2014. Os artigos foram selecionados, de maneira independente, por dois pesquisadores diferentes, tendo como base critérios de inclusão e exclusão previamente estabelecidos, com um índice Kappa de concordância de 0,86. A qualidade metodológica dos artigos revisados foi analisada.

**Resultados:**

a estratégia de pesquisa identificou 270 artigos; 25 artigos foram selecionados após a aplicação dos critérios de inclusão e exclusão, mas apenas 11 foram qualificados para a análise final. As substâncias envolvidas na ancoragem ortodôntica foram divididas em três grupos principais: osteoprotegerina (OPG), bisfosfonatos (BFs) e outras substâncias (OSs).

**Conclusões:**

diferentes substâncias são capazes de alterar o ciclo de remodelação óssea, influenciando na função dos osteoclastos e, portanto, na movimentação dentária. Sendo assim, essas substâncias podem ser utilizadas para promover o máximo de ancoragem e prevenir movimentos indesejados. A OPG foi a substância mais eficaz no bloqueio da ação dos osteoclastos, reduzindo os movimentos indesejados.

## INTRODUCTION

Orthodontic tooth movements are based on bone remodeling that occurs after the application of mechanical forces.^1^ At present, despite the efficacy of orthodontic techniques, there are a number of circumstances in which treatment efficiency might be improved by modulating the activity of osteoclasts, and therefore, bone turnover.^2^ Different drugs are able to alter the bone remodeling cycle, thus influencing tooth movement, as shown in different experimental models. Several drugs that modify osteoclasts function, such as bisphosphonates (BPs), anti-inflammatories and other molecules (OMs), have been used to prevent anchorage loss in Orthodontics.^3^^-^ 8 On the other hand, recent research suggests that biological modulators, able to inhibit osteoclasts, could be used to address these problems, thereby providing new adjunctive approaches to orthodontic treatment. This is the case of osteoprotegerin (OPG), a glycoprotein involved in bone metabolism that inhibits osteoclast differentiation and activation.^9^ The local delivery of OPG adjacent to anchorage teeth may provide a novel pharmacological approach in preventing undesired tooth movement.^10^ If undesirable tooth movement could be prevented with blockers of bone loss, orthodontic treatment could be less complicated and safer.

In the present study, we present a systematic review of the scientific literature that analyzes available experimental data about the local application of different drugs used to provide orthodontic anchorage.

## MATERIAL AND METHODS

Search criteria: An electronic search was conducted in PubMed-Medline, Scopus and Cochrane databases covering the period from 2000 to July 2014, and using the following keywords: bisphosphonates, osteoprotegerin and pharmacological anchorage, combined with orthodontic or tooth movement. Only studies associating pharmacological application for anchorage purposes were considered. The search was performed by two calibrated reviewers who independently applied the inclusion and exclusion criteria to every article with adequate concordance (Kappa index, 0.86; Landis and Koch, 1977).^11^ Disagreements between the two reviewers were discussed with a third reviewer for consensus. Articles wherein at least one of the reviewers felt that reflected the purpose of this study were fully reviewed. Selected article references were reviewed in order to extend the search for relevant articles.

Criteria for inclusion - The following criteria were taken into account:

1. Animal studies considering drugs as a new approach to provide orthodontic anchorage.
2. Experimental animal studies including at least one experimental group and one control group.
3. Minimum of six animals or samples per experimental group.
4. Local administration - delivery of substances.
5. Application of forces throughout orthodontic or orthopedic devices.
6. Appropriate data analysis.
7. English, Spanish, German or French languages.

Descriptive studies, case reports, case series, review articles, letters and articles that did not correspond with the aim of this review were excluded.

The initial selection of articles was based on title and abstract, with a review of the complete article whenever there was any doubt about its inclusion. Studies were classified and stored by main author, publication year, study design, sample size, type of substance and via of administration, amount of applied forces, tests conducted and conclusions.

The methodological quality of the selected papers was assessed using the method described by Iglesias-Linares et al,^12^ based on that proposed by Antczak et al and Jadad et al.^13^^,^ 14 The following characteristics were considered: sample size, previous estimation of sample size, validity of measurement taking methods, appropriate statistics, method error analysis, blinding of measurements, and loss of subjects/animals to the study. Quality was classified as low, medium and high.

## RESULTS

Search results: Search strategy yielded 270 titles/abstracts. After applying the inclusion/exclusion criteria, 245 papers (about 90%) were removed, primarily because they were reviews, case reports, human studies or unrelated to orthodontic anchorage. The remaining 27 articles (10%) were read entirely and divided into three groups based on the type of substance used to provide orthodontic anchorage. The BPs group comprised nine studies; further on, five papers were excluded: three due to systemic drug application,^6^^,^ 15 ^,^ 16 one because it did not state the type of BP used^17^ and one because the experimental group size was too small.^18^ The OPG group comprised five studies, but three were excluded because local application of molecule was not used.^8^^,^ 19 ^,^ 20 The OMs group comprised 13 articles of which seven were rejected because the via of administration was intragastric (five cases),^21^^-^ 25 intraperitoneal (one case),^26^ or intramuscular,^27^ and one due to the small size of the experimental groups.^28^

**Figure 1:**
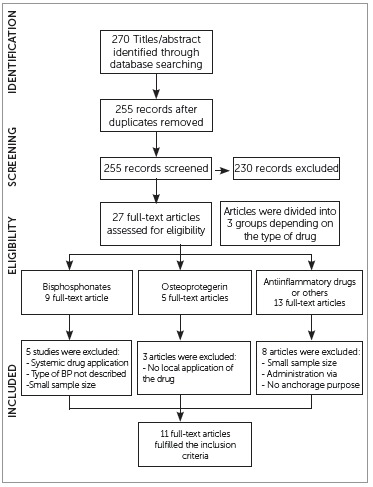
- PRISMA flow chart showing the process of study inclusion.

After careful analysis was carried out, 11 articles fulfilled all criteria for inclusion in the review (Fig 1).

Quality assessment: The eleven articles selected were based on animal studies; only two were considered to have high methodological quality, four were rated as of medium quality and five articles were of low quality. The main quality defects were inadequate size of study sample subgroups, absence of method error analysis and absence of blinding in measurements. The results of quality analysis are presented in Table 1.

Orthodontic anchorage: There is general consensus in the selected papers that orthodontic tooth movement is reduced after administration of the substances included in the present analysis. This lends support to the clinical use of these compounds in preventing undesired movements. Results for each type of drug under consideration are shown from Table 2 toTable 4.

**Table 1:** - Quality assessment of the articles included in the review.

**Molecules used for anchorage**	**Article**	**Sample size**	**Predetermined sample size**	**Measurement methods**	**Appropriate statistics**	**Method error analysis**	**Blinded measurements**	**Loss of animals to the study**	**Quality**
BPs	Liu et al^3^	Suitable	No	Suitable	Yes	No	No	No ($)	Low
	Choi et al^29^	Suitable	No	Suitable	Yes	No	No	No ($)	Low
	Ortega et al^7^	Suitable	Yes	Suitable	Yes	Yes	Yes	No	High
	Toro et al^30^	Suitable	Yes	Suitable	Yes	Yes	No	No ($)	Medium
OPG	Keles et al^31^	Small	No	Suitable	Yes	No	No	No	Medium
	Dunn et al^32^	Suitable	No	Suitable	Yes	Yes	Yes	No	High
OMs	de Carlos et al^4^	Small	No	Suitable	Yes	No	Yes	No	Medium
	de Carlos et al^5^	Small	No	Suitable	Yes	No	Yes	No	Medium
	Gurton et al^33^	Suitable	No	Suitable	Yes	No	No**	No*	Low
	Mermut et al^34^	Small	No	No	Yes	No	No**	No*	Low
	Chae et al^35^	Small	No	Suitable	Yes	No	No**	No*	Low

* Loss of animals or devices not clearly specified.

** No blinded measurements specified.

**Table 2:** - Articles related to bisphosphonates enclosed in the present review according to the inclusion/exclusion criteria.

**Author**	**Study design**	**Aim of the study**	**Sample size**	**Conclusion**
Choi J, et al^29^Korea 2010	Case control	Evaluation of the short-term effects of clodronate on early alveolar bone remodeling and root resorption related to orthodontic tooth movement.	54 sex-matched Wistar rats allocated into 3 groups of 18 rats: » 2.5 mmol/L clodronate, » 10 mmol/L clodronate » Control group	Clodronate inhibits bone resorption induced by orthodontic force. Both clodronate groups show significantly less tooth movement than controls, although the inhibition is greater in 10 mmol/L.
Liu L, et al^3^Japan 2004	Case control	Evaluation of the effect of local administration of clodronate on tooth movement.	26 male Wistar rats Split-mouth design: » 2.5 mM clodronate experimental side; vehicle contralateral side » 10 mM clodronate experimental side; vehicle contralateral side » 40 mM clodronate experimental side; vehicle contralateral side	Clodronate strongly inhibits orthodontic tooth movement.
Ortega A, et al^7^USA 2012	Case control	Evaluation of the use of local application of zoledronate to avoid loss of anchorage during extraction space closure in rats.	30 rats into 2 groups: » 15 control rats (vehicle) » 15 experimental rats (16 mg of zoledronate)	A single, small, locally applied dose of zoledronate was sufficient to provide maximal anchorage in extraction space closure.
Toro E, et al^30^Gainesville USA 2013	Case control	Evaluation of the effectiveness of Bis-enoxacin (BE) in the inhibition of bone resorption and orthodontic tooth movement.	30 Sprague Dawley into 3 groups: » Control: 10 rats (vehicle) » 2.5 mg/kg BE: 10 rats » 1 mg/kg Aledronate: 10 rats	BE inhibits osteoclast formation and bone resorption.
Keles A, et al^31^Boston USA 2007	Case control	Evaluation of the efficacy of pamidronate*versus* osteoprotegerin (OPG) in the inhibition of bone resorption and tooth movement.	18 C57Bl/6 male mice into 3 groups of 6 mice: » Control group (vehicle) » 5 mg/kg^-^ ^1^ pamidronate » 10 mg/kg^-^ ^1^ OPG	OPG results in a more powerful inhibitor of osteoclast recruitment and activity than pamidronate, with a reduction of osteoclasts number of 95% and tooth movement of 77%.

**Table 3:** - Articles related to osteoprotegerin enclosed in the present review according to the inclusion/exclusion criteria.

**Author**	**Study design**	**Aim of the study**	**Sample size**	**Conclusion**
Dunn M, et al^32^Michigan, USA 2007	. Case control	Assessment of OPG in regulating mechanically induced bone modeling in a rat model of orthodontic tooth movement.	39 male Sprague-Dawley rats » 9 rats without appliances: (3 rats without injections, 3 rats with vehicle and 3 rats with high-dose OPG) » 30 rats with orthodontic forces: (10 rats with 0.5 mg/Kg OPG-Fc, 10 rats with 5.0 mg/Kg OPG-Fc and 10 rats with vehicle)	Local delivery of OPG-Fc inhibits osteoclastogenesis and tooth movement at targeted dental sites.
Keles A, et al^31^Boston USA 2007	Case control	Evaluation of the efficacy of pamidronate*versus* osteoprotegerin (OPG) in the inhibition of bone resorption and tooth movement.	18 C57Bl/6 male mice into 3 groups of 6 mice: » Control group (vehicle) » 5 mg/kg-1 pamidronate » 10 mg/kg-1 OPG	OPG results in a more powerful inhibitor of osteoclast recruitment and activity than pamidronate, with a reduction of osteoclasts number of 95% and tooth movement of 77%.

**Table 4:** - Articles related to other molecules (OMs) enclosed in the present review according to the inclusion/exclusion criteria.

**Author**	**Study design**	**Aim of the study**	**Sample size**	**Conclusion**
De Carlos F, et al^4^Spain, 2006	Case control	Comparison of the effect of Diclofenac and Rofecoxib on the inhibition of dental movement in rats.	42 male Wistar rats in 6 experimental groups: » 3 groups subjected to a 50-g coil-spring (Diclofenac, Rofecoxib, control) » 3 groups subjected to a 10-g coil-spring (Diclofenac, Rofecoxib, control)	Both drugs significantly inhibit dental movement, partially in the case of Rofecoxib and totally in the case of Diclofenac.
De Carlos F, et al^5^Spain, 2007	Case control	Comparison of the effect of Rofecoxib, Celecoxib, and Parecoxib on the inhibition of dental movement in rats.	28 male Wistar rats in 4 groups subjected to 50-g force derived from a coil-spring: » 5 rats received Rofecoxib » 6 rats Celecoxib » 5 rats Parecoxib » 12 control rats	Rofecoxib inhibit tooth movement while Celecoxib and Parecoxib do not affect orthodontic movement.
Mermut S, et al^34^Ankara, Turkey 2007	Case control	Determination of the effects of IFN- ɣ on bone during orthodontic tooth movement.	30 male Sprague Dawley rats in five groups. (6 rats per group) » 0.01 µL IFN-ɣ dose » 0.02 µL IFN-ɣ dose » 0.05 µL IFN-ɣ dose » vehicle solution control group » only orthodontics control group	IFN- ɣ seems involved in bone remodeling during orthodontic tooth movement, which strongly suppresses osteoclastogenesis.
Gurton AU, et al^33^Ankara, Turkey 2004	Case control	Comparison of the effects of PGI2 and TxA2 analogs and inhibitors on orthodontic tooth movement.	150 male Sprague-Dawley rats in five equal groups, subdivided into three subgroups (SGs) depending on the concentration. » Iloprost (PGI2 analog) » indomethacin (PGI2 inhibitor), » U 46619 (TxA2 analog), » imidazole (TxA2 inhibitor) » control group	Indomethacin and Imidazole decrease the rate of tooth movement at high concentration, although there is no statistically significant difference between their inhibitory effects.
Chae HS^35^Seoul, Korea 2011	Case control	Evaluation of the effects of antioxidants on the expression of pro-inflammatory cytokines in human periodontal ligament fibroblasts (PDLFs) Evaluation of the effects of these antioxidants on the rate of orthodontic tooth movement in rats.	Two different assessments: Mechanical compression and induced hypoxia applied to human PDLFs exposed to 10 nM Resveratrol or 20 nM NAC orthodontic forces applied to 12 rats in a split-mouth design » 6 rats: experimental side treated with Resveratrol, contralateral side with vehicle solution » 6 rats: experimental side treated with NAC, contralateral side with vehicle solution	Antioxidants decrease the expression of pro-inflammatory cytokines in human PDLFs induced by mechanical compression and hypoxia *in vitro*. NAC delays orthodontic tooth movement in rats. Antioxidants may have the potential to retard orthodontic tooth movement.

## DISCUSSION

This systematic review was carried out to assess the effectiveness of different substances used to provide orthodontic anchorage. After exhaustive search and comprehensive evaluation, 11/270 articles were analyzed and categorized according to their methodological quality as low, medium and high. Due to the heterogeneity of the molecules found in the literature, three different groups were considered and analyzed separately.

The articles related to the use of BPs to provide orthodontic anchorage were included in the first group. BPs are potent bone resorption inhibitors frequently used to treat bone metabolism disorders, such as Paget disease, osteoporosis and bone metastases. Essentially, these drugs are internalized into osteoclasts, leading to inhibition of bone resorption and induction of osteoclasts apoptosis.^36^ Due to the wide range of information available about the action of these drugs and their ability to interfere in osteoclast activity and, thus, tooth movement, their use as pharmacological anchorage agents has been more referred in the literature than other drugs.^12^ Nevertheless, after applying the inclusion/exclusion criteria of the present review, only four articles were selected in this group. All of them were case-control studies carried on rats or mice. Choi et al^29^ used two different concentrations of clodronate and assessed alveolar bone remodeling and root resorption. This study showed significantly decreased root resorption with new bone formation, especially in the lower third of the roots; they also observed dose-dependent reduction of molar movement. These data agree with those provided by Liu et al^3^ who also recommended the local use of clodronate due to its anti-inflammatory properties that may be helpful in the treatment of increased bone resorption, such as periodontitis. This research defends the local application of BP in order to minimize potential systemic effects. Other studies focused on the analysis of the effects of BP on orthodontic anchorage. Zoledronate was applied locally in the extraction site of first molars and in second molars that were going to be protracted, resulting in significant reduction of tooth movement associated to bone preservation and fill.^7^ The effect of pamidronate was analyzed by Keles et al.^31^ The results of this study demonstrate tooth movement inhibition with a reduction in osteoclasts on the compression side.

The second group of molecules under consideration consisted of OPG. It is a soluble protein that inhibits the binding of receptor-activator of nuclear factor-B ligand (RANKL) to its cognate receptor, and prevents osteoclasts differentiation and activation. Dunn et al^32^ used two different doses of recombinant fusion protein OPG-Fc in a rat model and observed that this resulted in reduced molar movement and reduction in the number of osteoclasts. In comparing the effectiveness of OPG or BPs in tooth anchorage, Keles et al^31^ observed that OPG was more potent than pamidronate inhibition of tooth movement.

The third group included the OMs that are used in orthodontic anchorage. Anti-inflammatory drugs are frequently used to avoid pain and discomfort caused by orthodontic treatment, but these drugs could also produce decreased or slow-down tooth movement. De Carlos et al^4^ compared the effects of Diclofenac^(r)^ and Rofecoxib^(r)^, a conventional non steroideal anti-inflammatory and a COX-2 inhibitor, respectively. They found that both compounds are able to block tooth movement, being more potent the effect of Rofecoxib^(r)^ without significant statistical differences between groups. Thereafter, De Carlos et al^5^ also analyzed the effect of different anti-inflammatory substances on dental movement in a rat model. They found that Celecoxib^(r)^ and Parecoxib^(r)^, two compounds that specifically inhibit cyclooxygenase-2 (COX-2), did not affect tooth movement, while Rofecoxib^(r)^, which also affects COX-2, completely inhibited tooth movement in rats after 50-g force application. Inherent characteristics of these drugs, such as bioavailability, half live, etc., may account for discrepant effects of these compounds. Other drugs investigated in this group are the antioxidants. Chae et al,^35^ in their complete study, assessed the effects of antioxidants on the compression and hypoxia-induced production of pro-inflammatory cytokines in an *in vitro* evaluation of human periodontal ligament fibroblasts (PDLF). Moreover, based on the results obtained, they designed an animal study to assess the effect of these drugs on orthodontic tooth movement in rats. They used two different antioxidants; NAC, a precursor of glutathione that acts inhibiting the synthesis of pro-inflammatory molecules and Resveratrol, a naturally occurring phytoalexin present in grapes that has been shown to suppress osteoclast differentiation and promote osteoblast differentiation. These antioxidants demonstrated a decrease in the expression of proinflammatory, and NAC also produced a delay in orthodontic tooth movement in rats. Therefore, antioxidants, as it is suggested in these results, may have the potential to retard orthodontic tooth movement. On the other hand, Interferon-ɣ (IFN-ɣ) could result clinically useful for anchorage orthodontic control, as it has been observed in the study by Mermut et al.^34^ The authors found greater antiosteoclastic activity in the experimental groups compared to controls, thereby suggesting that IFN-ɣ is involved in bone remodeling during orthodontic tooth movement, acting as a strongly suppressor of osteoclastogenesis.

Several reviews have been published about the effects of different drugs on bone physiology and clinical side effects in Orthodontics.^37^^-^ 40 Even a recent review assessed the effects of medication on the rate of orthodontic tooth movement;^41^ however, to date, no review has been performed regarding the use of different drugs from a pharmacological anchorage approach. The present study is the first to assess the effectiveness of local application of three different groups of drugs that might be used to provide orthodontic anchorage by means of altering osteoclast function. As the desirable effects of these drugs should be local in order to obtain proper anchorage, this study only includes researches that involved local administration of these drugs. Thus, tooth movement of no anchorage teeth should be allowed and possible systemic effects avoided.

Due to the heterogeneity of the methods used to assess the effectiveness of drugs in experimental tooth anchorage, the results of this review cannot be analyzed together in a meta-analysis. Molar movement largely varied; the coil systems and the use of mini-screws, incisors or contralateral molars to maintain the coil were very different; as well as the time-points of analysis of the results ranged widely. Nevertheless, all studies analyzed agree with the fact that these drugs affect and reduce tooth movement. In the control group or side, tooth movement follows three different stages: a first phase of rapid movement, a lag phase and a progressive movement phase. In the experimental groups or side, the effects of the drugs are reveled at this late stage, which is considered to be due to bone resorption by osteoclasts. This suggests that their local administration inhibit bone resorption induced by orthodontic mechanical stress. Moreover, as it has been shown in Table 1, only a few articles in this study could be classified as high-quality, being most of them included in the medium and lower rank. Although these findings are promising, especially in the case of OPG, future research considering methodological quality is necessaries in order to determine the optimal dosage (quantity/frequency) and optimal administration methods that will allow greater amount of incisor retraction with the least anchor teeth movement, in addition to highly localized and long-term pharmacologic effects.

## CONCLUSIONS

Based on the findings extracted from the 11 selected articles, it can be concluded that: topical administration of BPs reduces tooth movement, which may be beneficial for anchorage procedures; also, osteonecrosis of the jaws was not found in any of the articles reviewed. Topical application of OPG reduces undesired tooth movements. OPG appears to be the most effective substance in blocking osteoclast function, being able to provide maximal anchorage after the application of orthodontic force. Topical application of anti-inflammatory drugs alters osteoclast function and, as a consequence, reduces tooth movement. Future studies are necessary to prove its effectiveness in humans.

In order to obtain better-quality scientific evidence, more prospective studies or randomized clinical trials are required on the use of these molecules in orthodontic therapy and their possible adverse effects.
